# Macrophage-Regulatory T Cell Interactions Promote Type 2 Immune Homeostasis Through Resistin-Like Molecule α

**DOI:** 10.3389/fimmu.2021.710406

**Published:** 2021-07-19

**Authors:** Jiang Li, Sang Yong Kim, Nancy M. Lainez, Djurdjica Coss, Meera G. Nair

**Affiliations:** Division of Biomedical Sciences, School of Medicine, University of California Riverside, Riverside, CA, United States

**Keywords:** splenomegaly, peritonitis, Treg, Th2 cytokine, M2 macrophage

## Abstract

RELMα is a small, secreted protein expressed by type 2 cytokine-activated “M2” macrophages in helminth infection and allergy. At steady state and in response to type 2 cytokines, RELMα is highly expressed by peritoneal macrophages, however, its function in the serosal cavity is unclear. In this study, we generated RELMα TdTomato (Td) reporter/knockout (Rα^Td^) mice and investigated RELMα function in IL-4 complex (IL-4c)-induced peritoneal inflammation. We first validated the RELMα^Td/Td^ transgenic mice and showed that IL-4c injection led to the significant expansion of large peritoneal macrophages that expressed Td but not RELMα protein, while RELMα^+/+^ mice expressed RELMα and not Td. Functionally, RELMα^Td/Td^ mice had increased IL-4 induced peritoneal macrophage responses and splenomegaly compared to RELMα^+/+^ mice. Gene expression analysis indicated that RELMα^Td/Td^ peritoneal macrophages were more proliferative and activated than RELMα^+/+^ macrophages, with increased genes associated with T cell responses, growth factor and cytokine signaling, but decreased genes associated with differentiation and maintenance of myeloid cells. We tested the hypothesis that Rα^Td/Td^ macrophages drive aberrant T cell activation using peritoneal macrophage and T cell co-culture. There were no differences in CD4^+^ T cell effector responses when co-cultured with RELMα^+/+^ or RELMα^Td/Td^ macrophages, however, RELMα^Td/Td^ macrophages were impaired in their ability to sustain proliferation of FoxP3^+^ regulatory T cells (Treg). Supportive of the *in vitro* results, immunofluorescent staining of the spleens revealed significantly decreased FoxP3^+^ cells in the RELMα^Td/Td^ spleens compared to RELMα^+/+^ spleens. Taken together, these studies identify a new RELMα regulatory pathway whereby RELMα-expressing macrophages directly sustain Treg proliferation to limit type 2 inflammatory responses.

## Introduction

Macrophages are a dominant resident population within the peritoneal cavity with critical immune surveillance and homeostatic functions (1). As sentinels, they are rapid responders to microbial invasion resulting from injury of the serous organs, such as the spleen, liver and intestinal tract, and can be mobilized to traffic to the injured organ and mediate tissue repair (2). Peritoneal macrophages also perform homeostatic functions to support innate B1 cells (3), clear debris and apoptotic cells (4, 5), and dampen inflammation (6–8). On the other hand, dysregulated peritoneal macrophage responses are associated with diseases including peritonitis, bacterial dissemination, and cancer metastases (9–12). Identification of peritoneal macrophage-derived factors and activation markers that cause beneficial or pathologic outcomes would provide insight into their biology and identify targets for treatment of serous cavity-associated disease.

Peritoneal macrophages, especially the monocyte-derived small peritoneal macrophages, express Resistin-like molecule α (RELMα) under homeostatic conditions (13). In type 2 cytokine-polarized environments such as helminth infection or *in vivo* IL-4 complex injection, RELMα expression is dramatically elevated reaching 100% expression by small and large peritoneal macrophages (14). RELMα, also known as FIZZ1 and HIMF, was originally identified as a highly secreted protein in the lung during allergic airway inflammation (15), however, it is now well-recognized that RELMα is pleiotropically expressed throughout the body, and a signature gene expressed by M2-polarized macrophages in response to multiple helminth infections (16, 17). RELMα expression is also triggered by other signals in addition to type 2 cytokines, for example by phagocytosis of apoptotic cells through scavenger receptors (18), or in response to hypoxia (19). Studies in pulmonary inflammation, hypertension and fibrosis, point to an inflammatory function for RELMα by promoting immune cell recruitment, fibroblast activation and proliferation associated with pathogenic fibrosis (20, 21). On the other hand, in response to tissue migratory helminth parasites, RELMα critically prevents fatal lung tissue damage, granulomatous inflammation, and promotes tissue repair (22–28). Downstream regulatory mechanisms for RELMα include limiting CD4^+^ T cell polarization, promoting anti-inflammatory responses, and mediating collagen cross-linking associated with tissue healing (26, 29, 30). RELMα also exhibits antibacterial properties by disrupting bacterial membranes for certain bacterial species (31).

Despite high expression levels of RELMα by peritoneal macrophages, whether RELMα affects the role of these cells in immune surveillance or homeostasis is unknown. In this study, we generated transgenic mice where RELMα is deleted and replaced with the TdTomato reporter protein (Rα^Td^) and investigated the consequence of RELMα deletion in a polarized type 2 cytokine environment caused by injection of IL-4 complexes. We first validated the Rα transgenic mice and demonstrated successful deletion of RELMα and expression of TdTomato protein, which had an equivalent expression pattern to RELMα. Next, we compared PBS and IL-4c injected Rα^+/+^ and Rα^Td/Td^ mice, where we identify a critical role for RELMα in limiting IL-4-induced peritoneal macrophage expansion, M1 macrophage activation, and splenomegaly. Gene expression analysis of sorted macrophages from Rα^+/+^ and Rα^Td/Td^ mice revealed that RELMα deficiency leads the induction of genes promoting T cell response, growth factor and cytokine signaling, but decreased genes associated with differentiation and maintenance of myeloid cells. Combining macrophage-T cell co-cultures, and investigation of *ex vivo* T cell responses, we further identify a role for macrophage-derived RELMα in promoting regulatory T cell proliferation and the production of IL-10 and GM-CSF. Together, results from these studies validate the utility of Rα^Td/Td^ mice to track RELMα expression and identify a dual role for RELMα in limiting type 2 cytokine immunopathology by cell-intrinsic effects on macrophages and regulatory T cells.

## Materials and Methods

### Mice

*Retnla^Td^* transgenic mice were generated by genOway (Lyon, France) by homologous recombination in C57BL/6 embryonic stem cells. *Retnla* Exon 2-4 was targeted using cre recombinase and Flp-mediated excision and replacement with the Td reporter gene, with a WPRE site (32) to enhance reporter expression and stability. The mice were crossed with the genOway proprietary cre-deleter mouse (pCMV driven cre) to generate constitutive Retnla^Td^ mice. Retnla^Td/+^ heterozygote mice were crossed with C57BL/6 mice to generate littermate homozygote (Td/Td) and WT (+/+) mice after three generations, then bred in-house. Arginase^YFP^ (Yarg) mice were available from Jackson labs. Mice were age matched (6 to 14 weeks old), sex-matched for experiments, and housed under an ambient temperature with a 12 hours light/12 hours dark cycle.

### IL-4 Complex (IL-4c) Injection

Mice were injected intraperitoneally (i.p.) with 2.5μg of recombinant mouse IL-4 (Peprotech, Rock Hill, NJ) complexed to 12μg 11B11 (Bio X Cell) in 100μl PBS or 100μl PBS (vehicle control) on days 0 and 2 and peritoneal cells and spleen were recovered at day 4, according to published studies (17).

### RNA Isolation and qPCR

Cells were washed twice with ice-cold PBS and lysed with RLT buffer. RNA was extracted by using the Aurum total RNA mini kit (Bio-Rad, San Diego, CA). cDNA was generated by iScript reverse transcriptase (Bio-Rad, San Diego, CA). RT-qPCR was performed with the Bio-Rad CFX Connect system using Bio-Rad CFX Manager 3.1 software. RELMα primers were purchased from Qiagen, Td Tomato primer sequence were (F: 5’-CCA CCT GTT CCT GGG GCA-3’, R: 5’-ACT CTT TGA TGA CGG CCA TGT-3’), and 18s primer sequences were (F: 5’-ACG GAA GGG CAC CAC CAG GA-3’, R: 5’-CAC CAC CAC CCA CGG AAT CG-3’)

### Immunofluorescence

Spleens were recovered and immediately immersed in 4% PFA. After 24 hours, tissue was removed from 4% PFA and incubated for 24 hours in 30% sucrose. For immunofluorescent staining, sections were incubated with rabbit anti-RELMα [2.5ug/ml; (Peprotech, Rock Hill, NJ)] or rabbit anti-TdTomato (Rockland Immunochemicals, Limerick, PA, USA), PE-anti CD3 (BioLegend, San Diego, CA) and FITC Rat anti-Foxp3 (eBioscience, Santa Clara, CA) overnight at 4°C. Sections were incubated with Cy5 anti-Rabbit fluorochrome-conjugated antibodies for 2 hours at room temperature (Abcam, Cambridge, Ma), then counterstained with DAPI (BioLegend, San Diego, CA). FoxP3^+^ cells were quantified by the ImageJ software.

### Flow Cytometry and t-SNE Analysis

Peritoneal cavity cells (PECs) were recovered in a total of 5 mL of ice-cold PBS. Splenic macrophage isolation were performed according to previous studies (33).Visceral fat was dissected and single cell dissociation and staining performed as previously described (34). For flow cytometry, cells were blocked with 0.6 µg Rat IgG and 0.6 µg α-CD16/32 (2.4G2) 5 min, stained for 25 min with antibodies to CD11b (M1/70), MHCII (M5/114.15.2), CD11c (N418), CD4(RM4-5), Ly6C(HK 1.4), Ly6G(1A8), CD19(1D3) and CD8(53-6.7) (all from BioLegend, San Diego, CA); SiglecF (E50-2440) (BD Bioscience, San Jose, CA); F4/80 (BM8) (eBioscience, Santa Clara, CA). Cells were analyzed on a Novocyte (ACEA Biosciences, San Diego, CA) or LSRII instrument (BD Bioscience, San Jose, CA) followed by data analysis using FlowJo v10 (Tree Star Inc.; Ashland, OR).** **t-SNE analyses were performed with FlowJo v10, involving concatenation of samples (5000 cells per biological replicate) from all groups before running the t-SNE analyses to generate plots consistent between groups. This was followed by analysis of separated groups for expression of the desired markers. The parameters used to run the t-SNE analyses are in **Supplementary Table 1**. Arg, Rα or TdTomato were excluded as parameters given that their expression was being analyzed, and they were negative in the some of the transgenic mouse groups.

### Cytokine Quantification

For sandwich ELISA, capture and biotinylated detection antibodies were used according to previously described protocols (25). IL-23, IL-1α, IFN-γ, TNF-α, MCP-1, IL-12p70, IL-1β, IL-10, IL-6, IL-27, IL-17A, IFN-β and GM-CSF were detected by the Mouse Inflammation Panel (13-plex) (BioLegend, San Diego, CA) and analyzed on the LSRII instrument (BD Bioscience) and LEGENDplex™ software.

### Splenocyte Stimulation

Spleens were harvested from PBS or IL4c treated mice at day 4. Single cell suspensions were generated from whole spleen, and red blood cells lysed with ACK lysis buffer. Cells were stimulated in 48 well plates at 5x10^6^ cells/well with 1μg/ml of α-CD3 and α-CD28 (eBioscience) as described previously (29). Supernatants were recovered at day 3 for cytokine measurement.

### Macrophage and Splenocyte Co-Cultures

Peritoneal cells from naïve Rα^+/+^ or Rα^Td/Td^ mice were recovered and treated *in vitro* with IL-4 (20ng/ml) or equivalent control PBS in complete DMEM media (Invitrogen, Gaithersburg, MD). After 24hrs, supernatants were recovered for RELMα ELISA, cells were washed with PBS to remove non-adherent cells, followed by recovery of adherent macrophages with TrypLE™ Express (Invitrogen, Gaithersburg, MD) and plated in a 96-well flat bottom plate at 2x10^4^ cells/well. In vivo-derived M2 macrophages were generated by one i.p. injection of IL-4c, recovery of the peritoneal cells, and F4/80 bead purification using MS columns with >90% purity (Miltenyi Biotec, Inc). Splenocytes were recovered from naïve Rα^+/+^ mice, and single cell suspensions prepared as above. Splenocytes were CFSE-labelled (5μM, 15 minutes) as previously described (29) (Invitrogen, Gaithersburg, MD), then added to the macrophages (Mac : Splenocyte 1:10) with 0.5μg/ml α-CD3 (5 replicate wells per condition). After 3 and 6 days, non-adherent splenocytes were recovered for flow cytometry analysis on the LSRII (BD Bioscience), and supernatants were recovered for cytokine measurement.

### Nanostring Gene Expression Analysis

Peritoneal macrophages (CD11b^+^F4/80^+^) were sorted with the MoFlo Astrios cell sorter (Beckman Coulter). 5000 macrophages from PBS mice or IL-4c-injected mice were lysed with 1/3 RLT buffer diluted with ddH_2_0 (Qiagen). Lysed cells were processed and quantified by the Myeloid Innate Immunity v2 panel according to manufacturer’s instructions (Nanostring). Gene expression analysis was conducted using the Advanced Analysis Nanostring software. Raw counts were normalized to internal controls (4 housekeeping genes, *Eef1g, Gusb, Oaz1* and *Rpl19*), then normalized transcripts with n>30 counts were included for analysis (a total of 309 out of 734 genes). The Nanostring Advanced Analysis algorithm generated biological pathway scores by extracting pathway-level information from a group of genes using the first principal component (PC) of their expression data (35). Pathway scores of Rα^+/+^ or Rα^Td/Td^ naive and IL-4c-injected mice were analyzed by an unpaired t-test and chosen pathways (p value ≤ 0.05) are represented as the difference in pathway score between the Rα^+/+^ or Rα^Td/Td^ groups (n=4/group). Differentially expressed (DE) genes (p ≤ 0.05) in each pathway were graphed as heatmaps (36).

### Statistical Analysis

Data are presented as mean ± SEM and statistical analysis was performed by Graphpad Prism 9 software. Data was assessed by one-way ANOVA followed by post-hoc Tukey’s test for multiple comparison, or by unpaired t-test for 2-group comparisons. For data collected over several time points, two-way ANOVA with post-Sidak multiple test was performed. *, p ≤ 0.05; **, p ≤ 0.01; ***, p ≤ 0.001. Experiments were repeated 2-4 times with n=2-8 per group for *in vivo* experiments, or 3-5 replicate wells for *in vitro* studies, apart from Nanostring gene expression analysis, which was performed once (n=2 for naïve and n=4 for IL-4c injected per group).

### Mouse and Data Availability

RELMα transgenic mice are available at MMRC repository (067014-UCD, https://mmrrc.ucdavis.edu).

## Results

### Generation and Validation of RELMα Transgenic Mice

**Figure 1:**
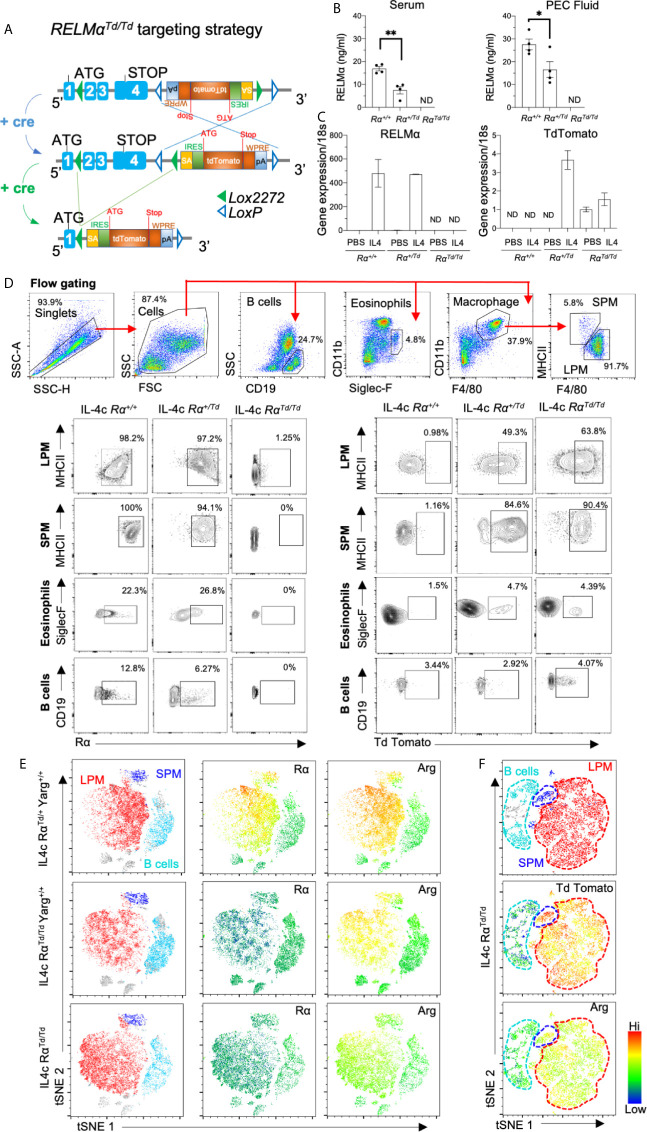
Validation of RELMα reporter transgenic mice. RELMα (Rα) or TdTomato (Td) reporter homozygote and heterozygote mice were intraperitoneally injected with PBS or IL-4c (2.5μg IL4/mouse) at day 0 and 2, then sacrificed at day 4. **(A)** Targeting strategy for RELMα deletion and replacement with Td reporter gene. **(B)** RELMα ELISA of serum and peritoneal fluid of 6–8 weeks mice. **(C)** RELMα and Td qRT-PCR of peritoneal cells treated *in vitro* for 24h with IL-4 (20ng/ml), presented as relative expression to PBS and normalized to 18s transcript. Rα expression was normalized to the PBS Rα^+/+^group (set at “1”) **(D)** Flow cytometry gating and analysis of large and small peritoneal cells (LPM and SPM), eosinophils and B cells from IL4c-injected mice for Td and RELMα protein. **(E, F)** t-SNE -guided analysis of peritoneal cavity cells subsets gated according to 1D (LPM, red; SPM, blue; B1 cell, cyan) for expression of Rα, Arg^YFP^ and Td employing Rα^Td^ and Yarg reporter mice. Results shown are combined from 2-3 independent experiments (n=4-12), apart from **(C, E)** which is one experiment, n=3 replicates. ND, not detected. *, p ≤ 0.05; **, p ≤ 0.01.

RELMα is a pleiotropic protein expressed by both immune and non-immune cells, and is detectable in the serum of naïve mice (16). In the serosal cavities, resident macrophages express RELMα in homeostatic conditions, however, RELMα expression is dramatically elevated in a type 2 cytokine environment such as helminth infection or IL-4c injection (17). We sought to address RELMα function in the peritoneal cavity by generating transgenic mice in which cre and flp recombinase mediates RELMα (exons 2-4) deletion and replacement with the TdTomato reporter protein (**Figure 1A**). To validate the targeting strategy and enable tracking of RELMα-expressing cells, these founder mice were crossed to a universal cre deleter mouse line so that RELMα expression can be tracked by Td reporter protein in heterozygote mice (Rα^Td/+^), while homozygote mice (Rα^Td/Td^) are used to investigate RELMα function. Quantification of RELMα protein in the serum and peritoneal cavity fluid indicated high levels of circulating RELMα under homeostatic conditions in Rα^+/+^ mice, detectable but significantly reduced RELMα in heterozygote (Rα^Td/+^) mice, and no RELMα in homozygote mice (Rα^Td/Td^) (**Figure 1B**). RELMα and Td mRNA levels were also quantified in adherent peritoneal cells from naïve mice treated *in vitro* with IL-4 (**Figure 1C**). Both Rα^+/+^ and Rα^Td/+^ macrophages had equivalent IL-4 induced RELMα expression. In IL-4 treated Rα^Td/Td^ and Rα^Td/+^ macrophages, Td expression was increased. Although the Td expression pattern was similar to RELMα in the heterozygote mice, the fold induction of Td was much lower than that of RELMα. This suggests differences in PCR efficiency, mRNA stability, or that deletion of RELMα has feedback consequences on the RELMα promoter and gene expression. We investigated if these potential differences in RNA levels were also observed at the protein level by flow cytometry. Intracellular RELMα and Td protein was evaluated by flow cytometry of peritoneal cells recovered from PBS or IL-4 complex (IL4c) injected mice (**Figure 1D** and **Figure S1A**). As expected, the main cellular sources of RELMα protein following IL4c injection were the small and large peritoneal macrophages (SPM and LPM) with >95% expression of RELMα in Rα^+/+^ and Rα^Td/+^. In the Rα^Td/+^ heterozygote mice, Td protein was induced by IL-4 with 50% expression in LPM and 85% expression in SPM. We also observed IL-4c induced expression of RELMα and Td by eosinophils and CD5^+^ B1 cells (**Figure 1D** and **Figure S1B**). Finally, we examined RELMα and Td expression in other organs such as the visceral fat and the spleen (**Figure S2A**), where we observed some IL-4 induced RELMα or Td expression by macrophages, but this was much lower in magnitude compared to the peritoneal cells.

To evaluate heterogeneity in serosal macrophage populations, we generated t-SNE plots on flow cytometry data from IL-4c-treated Arginase-YFP/Rα dual reporter mice (**Figure 1E**). The main subsets observed were LPM (red), SPM (blue) and B1 cells (cyan). When comparing heterozygote Rα^Td/+^Yarg^+/+^ and Rα^Td/Td^Yarg^+/+^, RELMα was expressed in SPM and LPM of heterozygote Rα^Td/+^ but not in homozygote Rα^Td/Td^ mice. Rα^Td/Td^ mice showed instead Td protein expression with similar expression pattern to RELMα. While SPM were a homogenous population with high level RELMα (or Td) expression, LPM exhibited more heterogeneity with mid and high level RELMα-expressing subsets (green vs yellow/red) (**Figure 1F**). In contrast, Arginase was more homogeneously expressed in both SPM and LPM (yellow/red). Together, these data validate effective RELMα deletion and replacement with TdTomato and indicate potential heterogeneity of RELMα compared to Arginase expression in the LPM. We also demonstrate that Rα^Td/+^ heterozygote mice have robust Td and RELMα protein expression, supporting the utility of this transgenic mouse model to track RELMα expression.

### RELMα^Td/Td^ Mice Suffer From Increased IL-4c Induced Pathology

**Figure 2:**
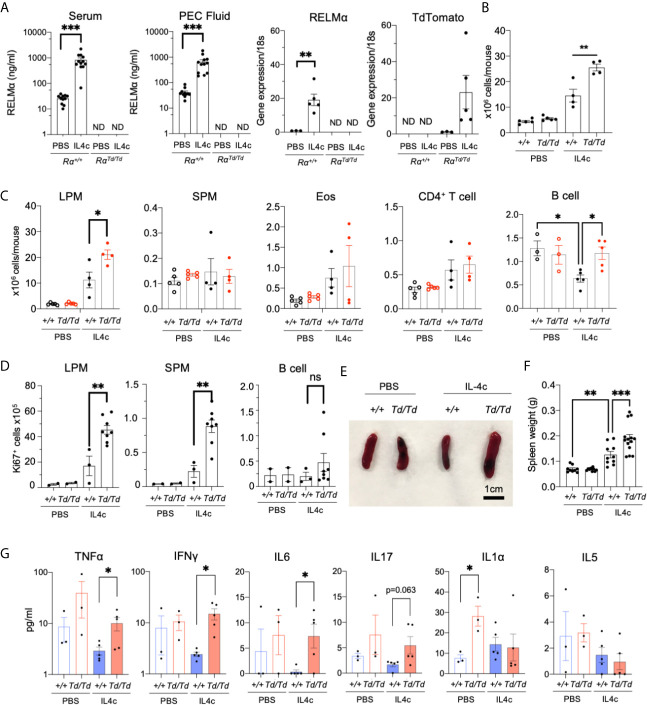
Increased IL-4-induced pathology in RELMα^Td/Td^ mice. PBS or IL4c-injected Rα^+/+^ or Rα^Td/Td^ mice were sacrificed at day 4 followed by evaluation of spleen and peritoneal cavity responses. **(A)** Peritoneal fluid and serum RELMα levels measured by ELISA, and RELMα and Td RNA was measured by peritoneal cell qRT-PCR as relative expression to PBS and normalized to 18s transcript. Rα expression was normalized to the PBS Rα^+/+^group (set at “1”) **(B, C)** Peritoneal inflammation and subset frequency evaluated by cell counts and flow cytometry analysis for small and large peritoneal macrophages (SPM and LPM), eosinophils, CD4^+^ T cells and B cells. **(D)** Peritoneal cell proliferation assessed by Ki67 staining. **(E, F)** Spleen size and weight were measured. **(G)** Serum cytokines were quantified. Results shown are representative of 3 independent experiments (n=4-8 per group). ND, not detected. *, p ≤ 0.05; **, p ≤ 0.01; ***, p ≤ 0.001.

Serosal macrophages that reside in the peritoneal cavity have important surveillance roles as sentinels for pathogen infections, but also regulate inflammation and can migrate to visceral organs to mediate repair (37). Peritoneal macrophages are main cellular sources with up to 100% RELMα expression following IL-4c injection, however, the function of RELMα in the peritoneal cavity has not been investigated. Wild-type (Rα^+/+^) or RELMα-deficient (Rα^Td/Td^) mice were injected with PBS or IL-4c. IL-4c injection led to significantly increased RELMα protein in the serum and RELMα mRNA in the peritoneal cells of Rα^+/+^ mice, while Td mRNA was significantly elevated in peritoneal cells of Rα^Td/Td^ mice (**Figure 2A**). As previously reported (14), IL-4c caused increased peritoneal cell numbers in Rα^+/+^ mice, but peritoneal inflammation was exacerbated in Rα^Td/Td^ mice (**Figure 2B**). Flow cytometric analysis revealed that LPM were the main cell-type affected by RELMα deficiency (**Figure 2C**). In the Rα^+/+^ mice, peritoneal B cell numbers were significantly decreased by IL4c treatment (**Figure 2C**), and further subsetting into CD5^+^ B1 cells and CD23^+^ B2 cells revealed that the decrease was significant in B2 cells (**Figure S2B**). In contrast, neither B1 nor B2 cells were reduced by IL-4c in Rα ^τd/Td^ mice, and B1 cells were significantly higher in IL-4c treated Rα^Td/Td^ mice compared to IL-4c treated Rα^+/+^ mice (**Figure S2B**), suggesting that RELMα is downstream of IL-4c mediated reduction in B cells. Other peritoneal cell subsets were not affected by IL-4c treatment nor RELMα deficiency. IL-4c induces significant LPM proliferation, therefore we evaluated Ki67 expression as a marker for proliferation. There was a significant increase in Ki67 positive LPM and SPM in IL-4c injected Rα^Td/Td^ mice but no changes in B cells (**Figure 2D**). RELMα-deficient mice also exhibited IL-4 induced splenomegaly, which was more severe than observed in wild-type mice (**Figures 2E, F**). This suggested an exacerbated response in RELMα deficiency, similar to the macrophage activation syndrome caused by sustained IL-4 exposure (38). Proinflammatory cytokine measurement in the serum revealed that Rα^Td/Td^ mice had increased circulating cytokines compared to Rα^+/+^ mice, with significant increases in IL1α under homeostatic conditions, and increased TNFα, IFNγ and IL-6 following IL-4 treatment, but no changes in circulating type 2 cytokine IL-5 (**Figure 2G**). We also performed the same cytokine bead array analysis on peritoneal lavage fluid but did not observe detectable cytokine levels. Together, these data reveal that RELMα critically mitigates IL-4-induced inflammatory effects including LPM and SPM proliferation, splenomegaly and systemic proinflammatory cytokine expression.

### RELMα Deficiency Leads to Dysregulated IL-4 Induced Myeloid Gene Expression Associated With T Cell Response, Growth Factor and Cytokine Signaling, and Myeloid Differentiation

**Figure 3:**
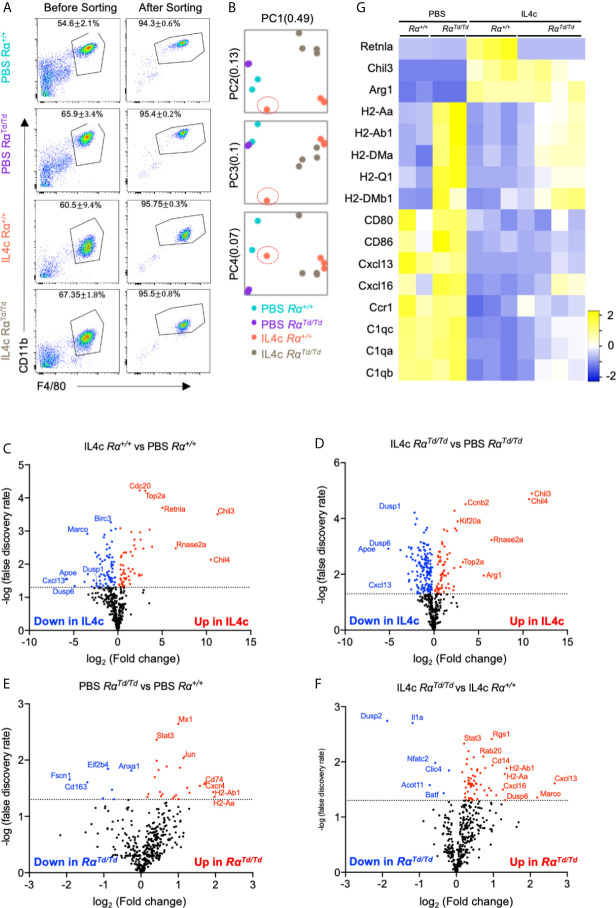
Gene expression analysis of peritoneal macrophages identify IL-4-induced and suppressed genes that are regulated by RELMα. Peritoneal macrophages were sorted from PBS or IL4c-injected Rα^+/+^ or Rα^Td/Td^ mice and analyzed by using the Nanostring myeloid gene expression panel. **(A)** Flow cytometry gating strategy and purity. **(B)** Principal component analysis demonstrates sample clustering. **(C–F)** Volcano plot of genes that are most changed in response based on IL-4c treatment **(C, D)** or genotype **(E, F)**. **(G)** Heatmap of canonical macrophage activation genes in different groups. Nanostring gene expression data are from a single experiment (n=2 for PBS and n=4 for IL-4c injected).

To identify mechanisms underlying RELMα regulation of peritoneal macrophage responses, gene expression analysis was performed in F4/80^+^CD11b^+^ peritoneal macrophages sorted from PBS or IL-4c treated Rα^+/+^ or Rα^Td/Td^ mice, using the Nanostring myeloid immunity panel (734 genes) (**Figure 3A**). Principal component analysis (PCA) demonstrated clustering according to genotype and treatment, with IL-4 treatment driving the greatest transcriptional differences regardless of genotype (**Figure 3B**). Out of four mice, macrophages from one IL-4c-treated Rα^+/+^ mice appeared as an outlier and clustered with the PBS-treated group (**Figure 3B**, red circle). Retrospective analysis revealed that this mouse had less RELMα in the PEC fluid, and lower peritoneal cell numbers likely because of ineffective IL-4c delivery (**Figure S3A**), therefore this sample was removed from gene expression analyses.

Investigation of the most differentially expressed genes indicated that Chil3/4 (Ym1/Ym2) and Rnase2a (Ear11) were highly upregulated by IL-4 for both mouse genotypes (**Figures 3C, D**). As signature M2 macrophage genes, RELMα and Ym1/2 are reported to have equivalent expression patterns, but Ym1 can also promote RELMα expression (24). Ear11 is an eosinophil-associated ribonuclease that is secreted by M2 macrophages and promotes neutrophil chemotaxis (39). Consistent with IL-4 induced resident macrophage proliferation, genes associated with the cell cycle (Top2a, Cdc20, Kif20a, Ccnb2) were upregulated. Consistent with an anti-inflammatory function for M2 macrophages, both Rα^+/+^ and Rα^Td/Td^ macrophages from IL-4c treated mice had reduced expression of genes associated with chemotaxis (Cxcl13, Cxcl14, Cxcl2), complement responses (C1qa, C1qb, C1qc) and innate immune activation (Birc3, CD80, CD86) (**Figures 3C, D, G**). Unexpectedly, the dual-specificity protein phosphatases (Dusp1, Dusp6), and Apoe were also suppressed by IL-4, although these have proposed anti-inflammatory and repair functions (40). These expression patterns likely reflect *in vivo* macrophage plasticity and the unique response of resident peritoneal macrophages to repeated treatment with IL-4, which may lead to negative feedback pathways for type 2 cytokine signaling. Overall, these genes were similarly induced or suppressed by IL-4 in both Rα^+/+^ and Rα^Td/Td^ mice, suggesting that these resident M2 macrophage activation programs occur even in the absence of RELMα.

**Figure 4:**
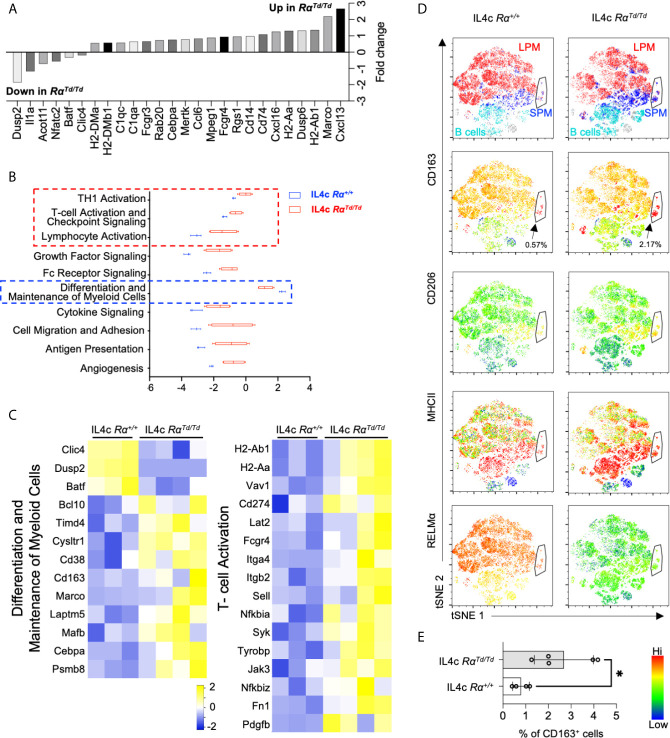
RELMα-deficient M2 macrophages exhibit a hyperactivated macrophage phenotype. Differential expression of genes associated with functional pathways was evaluated in macrophages sorted from IL-4c injected Rα^+/+^ or Rα^Td/Td^ mice. **(A)** Genes with highest Log2 fold change differences between IL4c injected Rα^+/+^ and IL4c Rα^Td/Td^ identified by Nanostring advanced analysis (p < 0.05). **(B)** Advanced pathway analysis performed on IL4c Rα^+/+^ and IL4c Rα^Td/Td^ macrophage RNA reveals significantly increased or decreased pathway scores (p<0.05). **(C)** Heatmap of differentially expressed genes within the functional pathways. **(D)** t-SNE-guided flow cytometry analysis of peritoneal cell subsets (LPM, red; SPM, blue; B1 cell, cyan). Black arrow and outline indicate a separate subset with SPM and LPM characteristics. Subsets were evaluated for expression of CD163, CD206, MHCII and RELMα. **(E)** Frequency of CD163^+^ macrophages in IL4c-treated Rα^+/+^ and Rα^Td/Td^ mice. *, p ≤ 0.05.

We next evaluated RELMα-regulated genes (**Figures 3E–G**). The most consistently upregulated genes in PBS or IL-4-induced Rα^Td/Td^ macrophages were MHC class II genes associated with antigen presentation (H2 genes, CD74), suggesting enhanced antigen presentation function by macrophages in RELMα-deficient mice even in homeostatic conditions. RELMα-deficient macrophages also had increased expression of chemokine/chemokine receptors (Ccl6, Cxcl13, Cxcl16) (**Figure 4A**). Cxcl13, involved in B1 cell maintenance (41), was the most upregulated gene in IL-4c induced Rα^Td/Td^ macrophages compared to Rα^+/+^ macrophages, consistent with the increased B1 cell numbers in RELMα-deficient mice. Conversely Dusp2, which negatively regulates cell proliferation (42, 43), was the most downregulated in the Rα^Td/Td^ macrophages (**Figure 4A**), consistent with their enhanced proliferation. Advanced pathway analysis (35) was performed to determine functional pathways that were significantly altered by RELMα following IL-4 treatment (**Figure 4B**). Consistent with upregulation of genes associated with macrophage hyperactivation, functional pathways that were significantly induced in RELMα-deficient macrophages involved enhanced T cell responses (Th1 activation, T-cell activation, antigen presentation). RELMα-deficient macrophages also induced genes associated with cytokine and growth factor signaling (Pdgfb, Jak3, Syk), which may have contributed to their increased expansion in response to IL-4 (**Figure 4C**). This increased macrophage proliferation and frequency in Rα^Td/Td^ mice may therefore result from dual effects of increased growth factor expression and responsiveness, and decreased expression of downregulatory checkpoints, such as Dusp2 and Batf (**Figure 4C**). Rα^Td/Td^ macrophages showed a reduction in genes associated with differentiation of myeloid cells (Mafb, Cebpa, Laptm5), which may reflect the enhanced proliferation rather than differentiation or maturation of these macrophages in the absence of RELMα.

Genes associated with angiogenesis (Fn1, Pdgfb) (44), phagocytosis (MerTK, Timd4, MHCII, C1q, CD16/32, Rab20 and Anxa1) (45–47), and the scavenger receptors (Marco, CD163) were increased in Rα^Td/Td^ macrophages (**Figure 4C**). This was consistent with the IL-4 treated Rα^Td/Td^ mice exhibiting characteristics of macrophage activation syndrome, associated with splenomegaly and erythrophagocytosis (38). To further analyze and validate some of these genes and their association with RELMα at the single cell and protein level, we use t-SNE mapping of flow cytometry data of peritoneal cells (**Figure 4D**). The t-SNE plots indicated the presence of a small subset that shared SPM and LPM characteristics (black arrow). This subset had the highest expression of CD163 and MHC class II, and co-expressed RELMα in the Rα^+/+^ mice. Further, it was increased by four-fold in the Rα^Td/Td^ mice, suggesting that RELMα expression by this subset may provide an autocrine negative feedback to limit its own expansion. Consistent with the Nanostring data, the MHCII^hi^ expressing subsets (**Figure 4D**, red) were expanded in the Rα^Td/Td^ macrophages, especially in the SPM and SPM->LPM subsets, consistent with the significantly increased MHCII MFI in SPM but not LPM in Rα^Td/Td^ mice (**Figure S2C**). however, there was heterogeneous distribution in LPM reflecting two functionally distinct LPM subsets in response to IL-4. Anti-CD163 surface staining confirmed the Nanostring data that Rα^Td/Td^ macrophages had significantly elevated CD163 expression in the SPM, LPM and the SPM to LPM cell subset, and was co-expressed with the M2 macrophage marker CD206 (**Figure 4D**). CD163 is a scavenger receptor for hemoglobin and is increased in M2 macrophages associated with hemophagocytic syndromes (48). Combined with the increased expression of genes associated with phagocytosis and scavenger functions, our observations that CD163^+^ M2 macrophages are significantly expanded in Rα^Td/Td^ mice (**Figure 4E**) point to a causal link between enhanced macrophage scavenging and the exacerbated IL-4 induced inflammation and splenomegaly in Rα^Td/Td^ mice (38, 49–51).

### RELMα-Expressing M2 Macrophages Support Regulatory T Cell Responses

**Figure 5:**
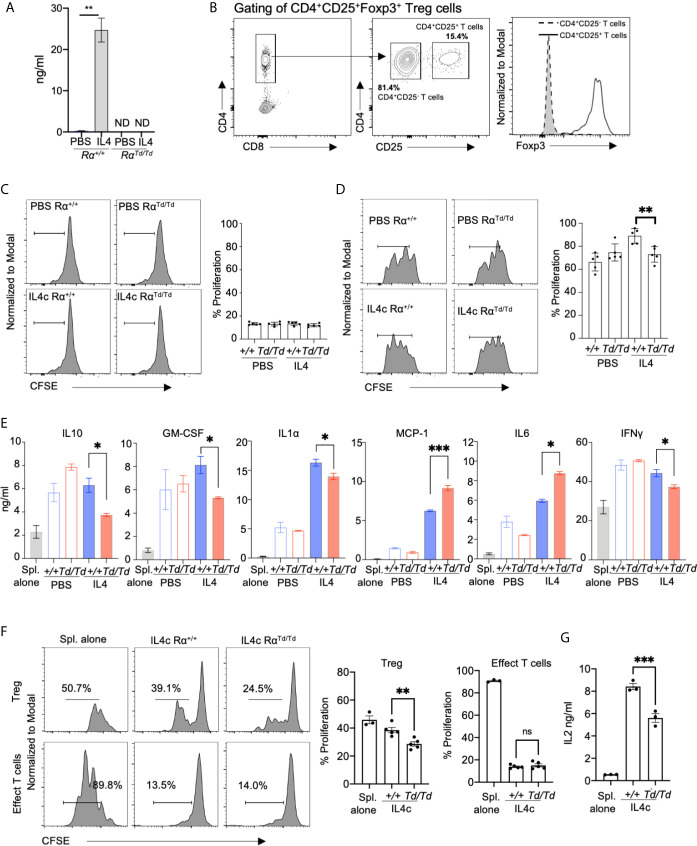
Macrophage-derived RELMα promotes regulatory T cell responses. Peritoneal macrophages from Rα^+/+^ or Rα^Td/Td^ mice were treated with PBS or IL-4 (20ng/mL) for 24h, washed then adherent macrophages were co-cultured for three days with CFSE-labeled splenocytes with anti-CD3 at a 1:10 (Mac : Splen ratio). **(A)** RELMα secretion by peritoneal macrophages treated *in vitro* with PBS or activated with IL-4. **(B)** T cell gating strategy. **(C, D)** Proliferation of CD4^+^CD25^-^ T cells **(C)** and CD4^+^CD25^+^FoxP3^+^ Tregs **(D)** when splenocytes are co-cultured with peritoneal macrophages treated *in vitro* with PBS or IL4 **(E)** Cytokine secretion measured at day 3 post-culture. **(F)** Proliferation of CD4^+^CD25^-^ T cells and CD4^+^CD25^+^FoxP3^+^ Tregs when splenocytes are co-cultured with F4/80+ peritoneal macrophages purified from *in vivo* IL-4c-treated mice. **(G)** IL-2 secretion measured at day 3 post-culture. ND, not detected; NS, not significant. *, p ≤ 0.05; **, p ≤ 0.01; ***, p ≤ 0.001.

Our *in vivo* data suggests that RELMα mice suffer from increased proinflammatory cytokine expression that is associated with enhanced macrophage activation, including increased expression of genes involved in T cell activation. We therefore investigated if macrophage-intrinsic RELMα dampens pro-inflammatory T cell responses using *in vitro* co-culture of peritoneal macrophages with splenocytes. Resident peritoneal macrophages from naive Rα^+/+^ or Rα^Td/Td^ mice were recovered and activated *in vitro* with IL-4, leading to significant RELMα secretion by Rα^+/+^ macrophages (**Figure 5A**). The macrophages were then recovered and co-cultured with CFSE-labeled splenocytes activated with anti-CD3. After 3 days of co-culture, only modest proliferation of effector CD4 T cells (CD4^+^CD25^-^) was observed (~15%) (**Figures 5B, C**), although robust proliferation was observed by day 6 (~70%) (**Figure S3C**). Although there were no differences in effector T cell proliferation, CD4^+^CD25^+^Foxp3^+^ regulatory T cells (Treg) exhibited robust proliferation (60-80%), which was significantly higher when co-cultured with IL-4 treated Rα^+/+^ macrophages compared to PBS treated Rα^+/+^ macrophages (**Figure 5D**). PBS-treated Rα^Td/Td^ macrophages supported equivalent Treg proliferation compared to PBS Rα^+/+^ macrophages, however IL-4 treated Rα^Td/Td^ macrophages were unable to enhance Treg proliferation (**Figure 5D**).

We evaluated the downstream effects of macrophage-Treg interaction by quantifying cytokine secretion. Macrophages or splenocytes cultured alone did not produce cytokines (**Figure 5E** and data not shown). Both PBS or IL-4 treated Rα^+/+^ macrophages-splenocyte co-cultures resulted in robust and equivalent secretion of IL-10, GM-CSF, and IFNγ, while IL-4-treated macrophages promoted enhanced secretion of IL-1α, MCP-1, IL-6. Co-cultures with PBS-treated Rα^Td/Td^ macrophages induced equivalent cytokine secretion to PBS-treated Rα^+/+^ macrophages, however, IL-4-treated Rα^Td/Td^ macrophages were unable to promote cytokines associated with Treg differentiation and function (IL-10 and GM-CSF). Instead, IL-4-treated Rα^Td/Td^ macrophages promoted secretion of MCP-1, IL-6. We also observed a reduction in IFNγ and IL-1α secretion in co-cultures with IL-4 treated Rα^Td/Td^ macrophages. Given that the co-cultures consisted of macrophages and splenocytes, the cellular source of the cytokines is unclear. Since the splenocytes are treated with anti-CD3, we conclude that most of the cytokines detected are directly from T cells, or indirectly from T cells activating other splenocytes or the peritoneal macrophages to produce cytokines.

We also investigated *in vivo*-derived M2 macrophages by IL-4c intraperitoneal injection, followed by recovery and purification of F4/80^+^ macrophages at day 1, and co-culture with anti-CD3 stimulated splenocytes (**Figure 5F**). Similar to the *in vitro*-derived M2 macrophages, co-culture with Rα^Td/Td^ macrophages led to significantly reduced Treg proliferation compared to Rα^+/+^ macrophages, with no significant effect on effector T cells. The Rα^Td/Td^ co-cultures also had significantly reduced IL-2 levels compared with the Rα^+/+^ macrophage co-cultures (**Figure 5G**), which may explain the reduced Treg proliferation. Together, this data suggests that macrophage-derived RELMα promotes Treg responses and suppresses myeloid expression of chemokines and proinflammatory cytokines, but has a mixed effect on T cell polarization and inflammasome activation.

### Dysregulated Splenic T Cell Responses and Reduced Regulatory T Cells in RELMα-Deficient Mice

**Figure 6:**
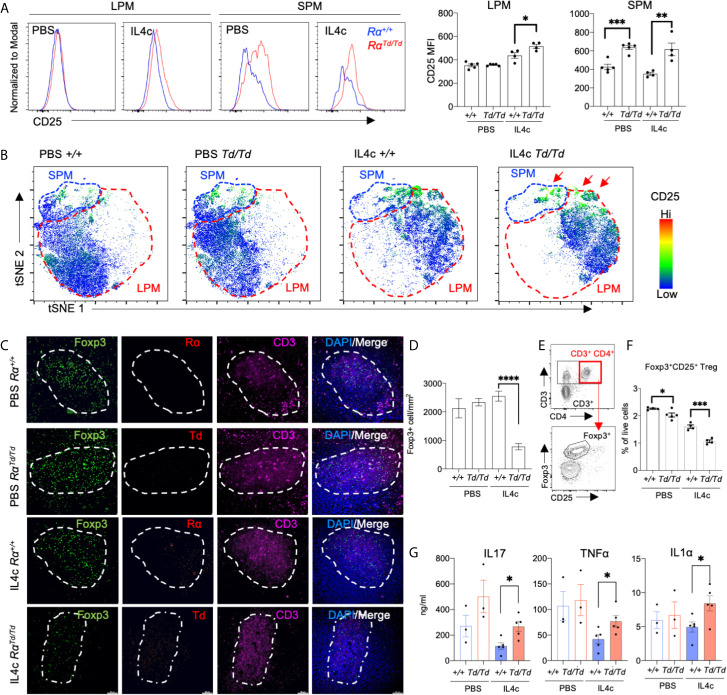
Impaired splenic regulatory T cell responses in RELMα-deficient mice are associated with increased inflammatory cytokines. Peritoneal cells and spleens from Rα^+/+^ or Rα^Td/Td^ mice treated with PBS or IL-4c were recovered for evaluation of macrophage and T cell responses. **(A)** CD25 expression in SPM and LPM. **(B)** t-SNE -guided gating of peritoneal cavity LPM and SPM and analysis of CD25. **(C, D)** Immunofluorescent staining for CD3, Foxp3, RELMα and Td was performed on spleen sections and Foxp3+ cells were quantified. Bar: 50μm. **(E)** Gating strategy for effector and regulatory splenic T cells. **(F)** Frequency of Tregs in spleen evaluated *ex vivo* by intracellular staining for Foxp3. **(G)** Cytokine secretion was quantified from splenocytes stimulated with αCD3/αCD28 for 72 hours. *, p ≤ 0.05; **, p ≤ 0.01; ***, p ≤ 0.001; ****, p < 0.0001.

Based on the co-culture results that demonstrated a direct effect of macrophage-derived RELMα in supporting Treg responses, we sought to determine the *in vivo* relevance of this novel regulatory function for RELMα. We therefore evaluated peritoneal macrophage and splenic T cell responses in PBS or IL-4c-treated Rα^+/+^ or Rα^Td/Td^ mice. We observed significantly increased CD25 expression in the Rα^Td/Td^ LPM and SPM (**Figure 6A**), which may provide one mechanism for limiting IL-2 availability to Tregs (52). t-SNE mapping showed that most IL-4c induced Rα^Td/Td^ SPM and a small subset of Rα^Td/Td^ LPM expressed CD25, compared to low expression in IL-4c induced Rα^+/+^ macrophages (**Figure 6B**). To validate the *in vitro* finding of impaired Treg responses in the absence of RELMα, we quantified Treg frequencies in the spleens of PBS or IL-4c-treated Rα^+/+^ and Rα^Td/Td^ mice. Immunofluorescent analysis of the periarteriolar lymphoid sheath of the spleen confirmed that IL4c-treated Rα^Td/Td^ mice had significantly reduced Foxp3^+^ cells (**Figures 6C, D**). Additionally, IL-4c treatment led to detectable RELMα protein expression in Rα^+/+^ mice and Td protein in Rα^Td/Td^ mice (**Figure 6C**), suggesting local effects of RELMα on the spleen. Flow cytometry analysis of the spleen also revealed significant reductions in CD4^+^CD25^+^ Tregs in IL4c-treated Rα^Td/Td^ mice compared to Rα^+/+^ mice (**Figures 6E, F**).

We next evaluated if the Treg deficiency in Rα^Td/Td^ mice was associated with dysregulated T cell polarization in the spleen. Anti-CD3 stimulation of splenocytes from IL-4c-treated Rα^Td/Td^ mice led to significantly increased secretion of IL-17A, TNFα and IL-1α compared to splenocytes from the counterpart Rα^+/+^ mice (**Figure 6G**). Combined, these *in vitro* and *in vivo* data reveal a previously unappreciated role for peritoneal macrophage-derived RELMα in mitigating IL-4 induced inflammation and immunopathology through promoting Treg responses and limiting proinflammatory macrophage and T cell responses.

## Discussion

As critical sentinels of the peritoneal cavity and visceral organs, the biology of peritoneal macrophages is increasingly being investigated (2, 53–56). These studies highlight the complexity and importance of these cells in response to infection and inflammation (55, 57), but also reveal their role in immune homeostasis (1, 2, 58, 59). Peritoneal macrophages follow similar activation pathways to other macrophage lineages, where M1 macrophages activated by IFNγ and TNFα have enhanced microbicidal or tumoricidal capacity and secrete high levels of pro-inflammatory cytokines and mediators (60), while IL-4 activated M2 macrophages reduce inflammation and contribute to tissue repair through secretion of IL-10 and TGF-β (61, 62). Although M2 macrophages have anti-inflammatory roles, dysregulation of these signaling pathways also induce inflammation and immunopathology (38), which we investigated using the *in vivo* model of IL-4c induced peritonitis. RELMα is a signature marker of small peritoneal macrophages under homeostatic conditions and is highly expressed by small and large peritoneal macrophages in a type 2 cytokine environment. However, the potential contribution of RELMα to peritoneal macrophage activation, function, or effects on other immune or resident cells are unknown. We addressed this question by generating RELMα transgenic mice and found that RELMα expressed by peritoneal macrophages acts back to limit macrophage proliferation and activation. Macrophage-derived RELMα was also critical to support regulatory T cell proliferation and function.

Genetically deficient RELMα mice have been previously investigated (28, 29, 31), and one study generated a RELMα-cre recombinase mouse line that enabled fate mapping of RELMα-expressing cells and diphtheria toxin-induced deletion of these cells (23). In the fate mapping RELMα-cre mice, any cell in which the *Retnla* promoter has been active at any time, will have constitutive reporter protein expression throughout its lifespan, therefore potentially overrepresenting RELMα expression. In contrast, the reporter mouse model described here can reflect temporal changes in *Retnla* promoter activity. Here, we validate its utility as a faithful reporter by side-by-side analysis of TdTomato reporter and RELMα mRNA and protein expression. Compared to the RELMα-cre mice, or other studies in helminth infection, we found that peritoneal macrophages were the dominant source of RELMα, while eosinophils and B1 cells only expressed modest levels of RELMα in response to IL-4. Our data is consistent with RNA-seq and Immgen datasets that evaluate naïve immune cell subsets (13), where small peritoneal macrophages are the highest RELMα expressors. Compared to other mouse models, these mice offer the potential to specifically delete RELMα within individual cells. Furthermore, this alleviates the need for diphtheria toxin, that causes apoptosis and can have pathologic consequences independent of RELMα function. In this study, we validated our transgenic mice by crossing them to a universal cre-deleter mouse line, however, this transgenic mouse model provides the valuable opportunity in future studies to delete RELMα in specific cell-types.

Consistent with other studies demonstrating a protective and anti-inflammatory role for RELMα, we show that RELMα is only expressed in naïve small peritoneal macrophage but is expressed by small and large peritoneal macrophages in IL-4-induced peritonitis. Within the peritoneal cavity, the main cell target of RELMα was the large peritoneal macrophage (LPM), where RELMα limited LPM proliferation and activation. This suggests that the same cell-type that produces RELMα is also its target, suggesting a macrophage-intrinsic negative feedback loop. Since IL-4 drives significant expansion of peritoneal resident macrophages, it may be important for immune homeostasis and energy conservation to have internal feedback mechanisms, such as RELMα, to keep this process in-check. For instance, sustained IL-4 exposure leads to immunopathology such as the macrophage activation syndrome, where splenomegaly is observed (38). The treatment regime in our studies involved only two IL-4c injections, however, Rα^Td/Td^ mice had already begun to exhibit immunopathologic features such as splenomegaly.

Gene expression analysis of the peritoneal macrophages from wild-type or RELMα-deficient mice indicated dual effects for RELMα in limiting proliferation and promoting survival, while also regulating macrophage polarization. RELMα-deficient macrophages had reduced expression of checkpoint inhibitors Dusp2, Batf (43, 63), but increased expression of anti-apoptotic signals such as Bcl2, Ctss and Pim2 (**Figure S3B**) (64, 65). RELMα-deficient macrophages exhibited increased genes associated with functional myeloid pathways compared to wild-type macrophages, suggesting overall enhanced myeloid activation. These included increased expression of Cd74, Cd14, Mpeg1, Ccl6 and MHCII, increased complement factors associated with the coagulation cascade (C1qa, C1qb, C1qc), and higher expression of phagocytic and scavenger receptors (Mertk, Cd32, Cd16, Marco). Most of RELMα-deficient macrophages upregulated genes associated M1 macrophage activation and antimicrobial function, including Cybb (66), Ifnar2 (67), Birc3 (68), Ccr5 (69) and Cd84 (70) (**Figure S3B**). In contrast, RELMα deficiency did not affect expression of other M2 macrophage signature genes such as Ym1 or Arginase, suggesting a regulatory effect of RELMα through inhibiting M1 activation, rather than promoting M2 activation.

Although gene expression analysis was performed on bulk-sorted peritoneal macrophages and did not distinguish monocyte-derived SPM from resident LPM, RELMα deficiency resulted in a heterogeneous macrophage phenotype with SPM and LPM markers. These included increased expression in RELMα-deficient macrophages of LPM-specific genes such as Timd4 and Cxcl13, but also SPM-associated genes MHCII, CD62L (Sell), CD38 and CD74 (71, 72). IL-4c-induced peritonitis has been previously shown to be caused by resident LPM proliferation rather than the recruitment of blood monocytes (17, 73). In our studies both Rα^Td/Td^ SPM and LPM showed evidence of increased proliferative capacity with elevated Ki67 expression compared to Rα^+/+^ macrophages, yet we did not observe significantly increased SPM numbers. It is possible that in the RELMα^-/-^ environment, SPM were transitioning to LPM, as has been reported in inflammatory environments (71–73). Indeed, the absence of RELMα led to increased circulating inflammatory cytokines including TNFα, IFN*γ*, IL-6 and IL-1α, and exacerbated splenomegaly. Gene expression analysis revealed increased genes associated with growth factor signaling and angiogenesis (e.g. Pdgfb, Ncf2 and Fn1) in the RELMα-deficient macrophages, which could have contributed to the splenomegaly.

The main regulatory effects of RELMα in limiting inflammation and immunopathology were observed following IL-4 treatment, however, MHCII genes (H2-Aa, H2-Ab1 and H2-DMa) were consistently elevated in the RELMα-deficient macrophages in both PBS and IL-4c treatment, suggesting a potential effect of RELMα on antigen presentation in a homeostatic or type 2 cytokine environment. To investigate the role of macrophage-intrinsic RELMα in T cell responses, we performed splenocyte co-cultures with peritoneal macrophages from wild-type or RELMα-deficient mice. RELMα deficiency had no significant effect on effector T cell responses, however, was unable to support optimal regulatory T cell proliferation. The direct mechanism underlying this defect is unclear, however, cytokine quantification revealed decreased Treg-associated cytokines GM-CSF (74) and IL-10, and conversely increased IL-6 in RELMα-deficient macrophage co-cultures. These co-culture findings were supported by the *in vivo* studies, where there was reduced Treg frequency in the spleen, but enhanced Th17 cell responses. RELMα-deficient macrophages had increased expression of the IL-2R (CD25), suggesting that they may limit IL-2 availability to the Tregs (75, 76), which was supported by our finding that IL-2 levels were significantly reduced in RELMα-deficient macrophage co-cultures. However, further experiments are needed to functionally link CD25 expression with IL-2 consumption by RELMα-deficient macrophages. Also, future investigation of the Tregs is warranted, such as their ability to suppress naïve T cells, and how their function is altered by RELMα. Immunofluorescent staining validated Treg reduction in the spleen, which may have contributed to the splenomegaly by removal of this regulatory brake. Macrophages in the spleen express RELMα, therefore splenic macrophage function may be similar to what was observed for the peritoneal cavity macrophages. An alternative possibility is that peritoneal macrophages migrate to the spleen, as prior studies observed peritoneal macrophage migration to other organs such as the liver in response to injury (2). Previous studies used bone marrow-derived macrophages or dendritic cells and *in vivo* helminth infection to address RELMα function in T cell polarization (28–30). Findings from these studies revealed many effects of RELMα on T cells, such as limiting Th2 cell polarization and promoting T cell-derived IL-10. Our studies support an immune regulatory role for RELMα on Tregs. However, we did not observe any differences in Th2 cell polarization, potentially because we interrogated the effect of RELMα on IL-4-induced responses, compared to the more complex outcomes and regulatory networks in helminth infection. Here, we further demonstrate the *in vivo* significance of resident peritoneal macrophages, which express significantly higher levels of RELMα than *in vitro* bone marrow-derived cells. We also identify a targeted effect of RELMα on promoting Treg proliferation with functional consequences to limit spleen immunopathology. Overall, these studies identify dual effects of macrophage-intrinsic RELMα in limiting macrophage activation while supporting Treg responses with the overall effect of limiting type 2 cytokine-mediated immunopathology. Investigation of this macrophage-Treg axis, and how it is influenced by RELMα, will be an important future direction to assess the biological significance of this interaction beyond IL-4c injection. Specifically, this immune regulatory role for RELMα in the peritoneal cavity may critically influence the outcome of type 2 cytokine-biased diseases such as helminth infection, injury and repair to visceral organs (77–80), but conversely may have impact in other settings where peritoneal macrophages and Tregs are detrimental such as in cancer metastases (81, 82).

## Data Availability Statement

The data has been uploaded to NCBI - accession number is GSE174606.

## Ethics Statement

The animal study was reviewed and approved by University of California Riverside Institutional Animal Care and Use Committee.

## Author Contributions

MN and JL conceptualized the study. JL and NL developed the methodology. JL, SK, and NL performed the investigation. MN and JL performed the formal analysis. MN and JL wrote the article. SK and DC edited the manuscript. MN and DC supervised the study. All authors contributed to the article and approved the submitted version.

## Funding

This research was supported by the UCR School of Medicine (to MN), and the National Institutes of Health (NIAID, R01AI153195 to MN). DC was supported in part by the National Institutes of Health (NICHD, R01HD091167). 

## Conflict of Interest

The authors declare that the research was conducted in the absence of any commercial or financial relationships that could be construed as a potential conflict of interest.
