# Supplementary material for: Macrophage-Regulatory T Cell Interactions Promote Type 2 Immune Homeostasis Through Resistin-Like Molecule α
Source: Front Immunol. 2021 Jul 19;12:710406. doi: 10.3389/fimmu.2021.710406 (PMC8327085; doi:10.3389/fimmu.2021.710406)
Supplement: Supplementary file 1 [file DataSheet_1.pdf]

Figure S1

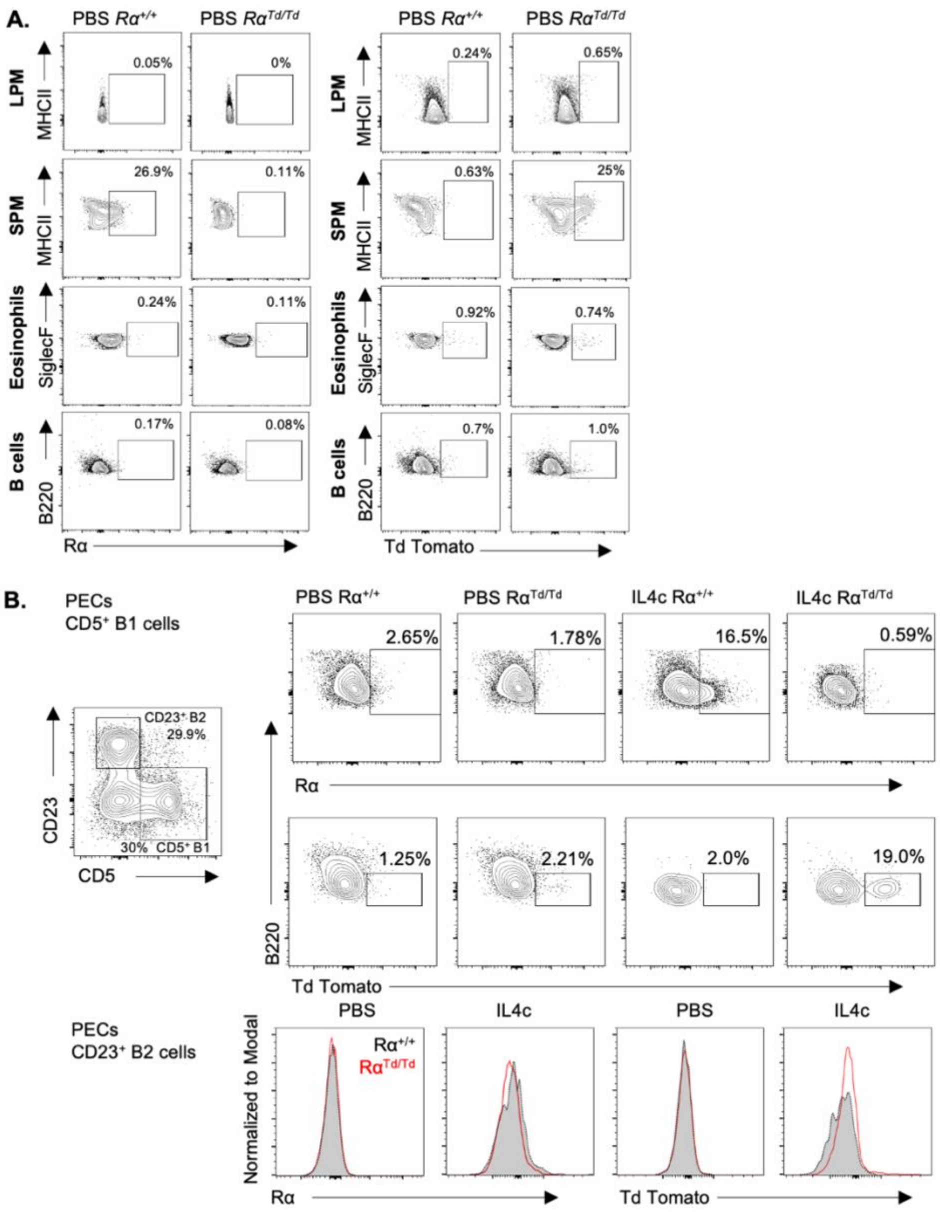

Supplementary Figure S1.

PBS or IL4c-injected R$\alpha^{+/+}$ or R$\alpha^{Td/Td}$ mice were sacrificed at day 4 post injection. **(A)** Flow cytometry gating and analysis of large and small peritoneal cells (LPM and SPM), eosinophils and B cells from PBS-injected mice for Td and RELMα protein. **(B)** RELMα and Td expression in peritoneal CD5^{+}B1 cells and CD23^{+} B2 cells from PBS or IL-4c-treated mice.

Figure S2

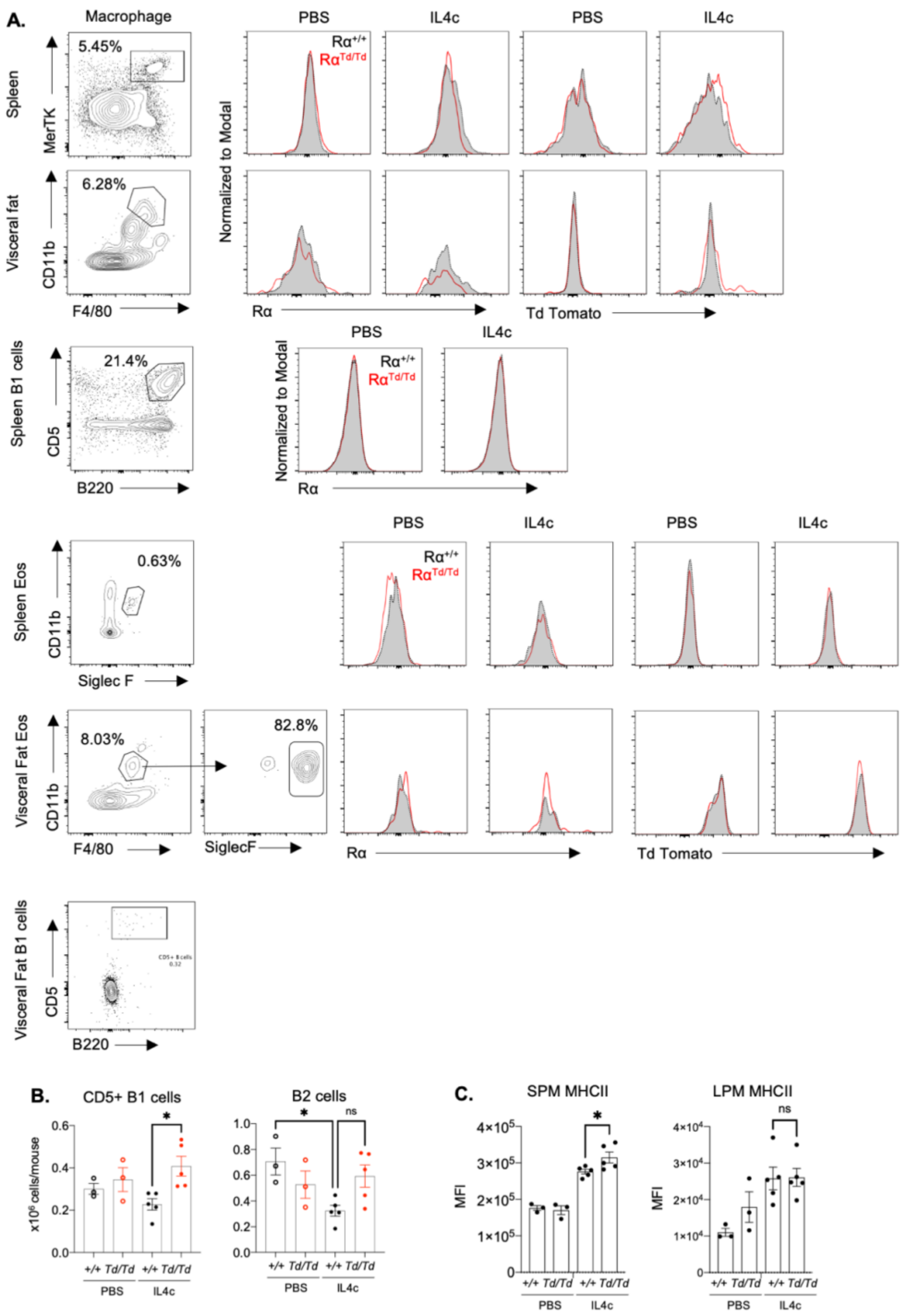

Supplementary Figure S2.

(A) Flow cytometry gating and analysis of RELMα and Td expression in spleen and visceral fat macrophages, B cells and eosinophils. **(B)** Peritoneal B1 and B2 cells numbers in PBS and IL-4c-treated mice. **(C)** MHC2 MFI of SPM and LPM isolated from PBS and IL-4c-treated mice.

Figure S3

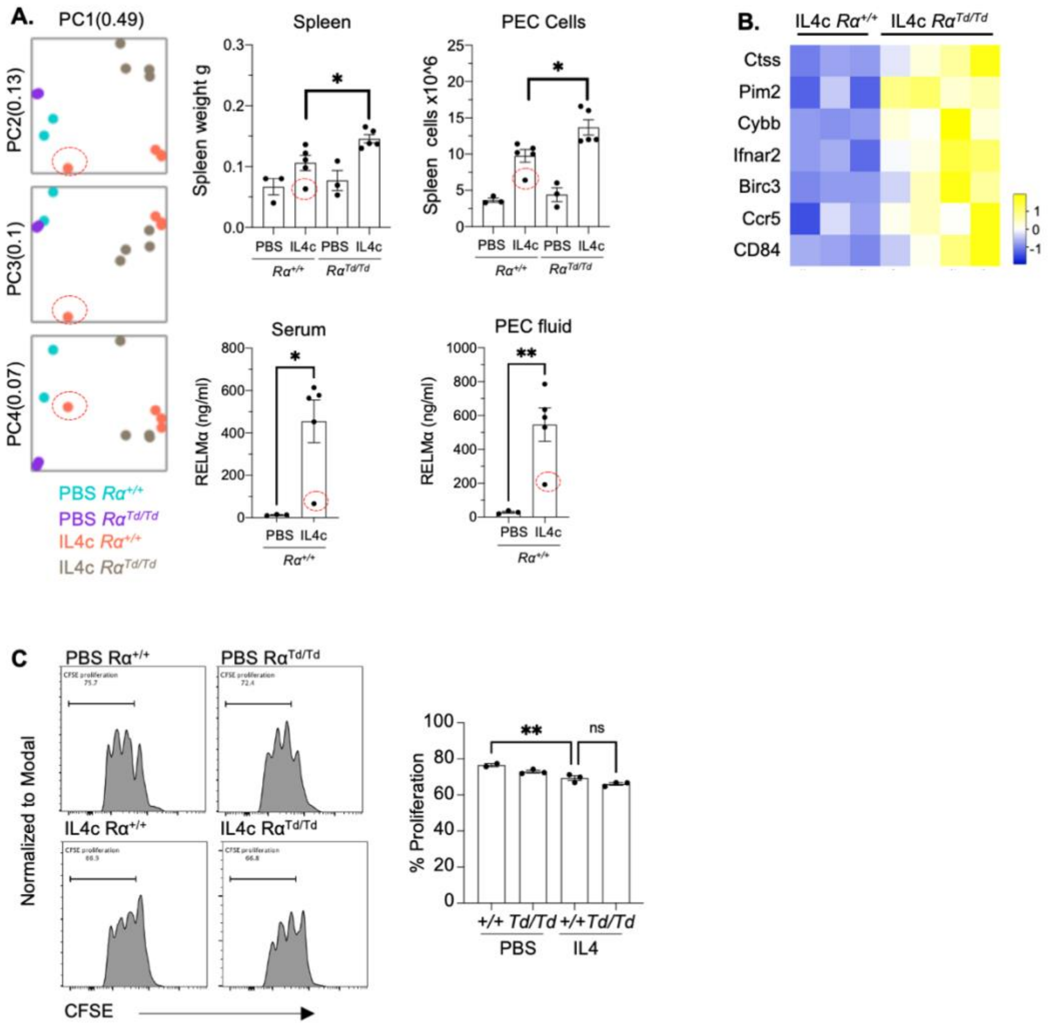

Supplementary Figure S3.

(A) Principal component analysis identifies one outlier in IL-4c-treated R$\alpha^{+/+}$ sample, which is confirmed by spleen weight and PEC cell counts, and serum and PEC RELMα levels (see red circle). **(B)** Heatmap of macrophage gene expression in different groups. (**C**) Effector T cell proliferation at day 6 post co-culture with naïve peritoneal macrophages treated *in vitro* with PBS or IL-4.

Table S1

Figure 1E

FITC	Arg
PE-Texas Red	SiglecF
PerCP-Cy5.5	CD4
PE-Cy7	F4/80
APC	Rα
Alexa Fluor 700	MHCII
A780	CD11b
BV421	Ly6G
BV510	CD19
BV605	CD11c

Figure 1F

FITC	Arg
PerCP	MHCII
PerCP Cy5.5	CD4
PE	TdTomato
PE-Texas Red	SiglecF
PE Cy5	CD11c
PE Cy7	F4/80

Figure 4

FITC	TNFα
PerCP	CD3
PerCP Cy5.5	IL-17
PerCP eFlour 710	B220
PE	Gata3
PE-Texas Red	SiglecF
PE Cy5.5	Foxp3
PE Cy7	Ly6c
APC	Rα
Alexa Flour 700	MHCII
APC Cy7	CD11b
Pacific Blue	CD163
AmCyan	Ly6G
Pacific Orange	CD8
Qdot 605	CD11c
Qdot 655	F4/80
BV711	CD4

Figure 6

FITC	CCR1
PE	CD25
PE-Texas Red	SiglecF
PerCP-Cy5.5	CD4
PE-Cy7	F4/80
APC	CD206
Alexa Fluor 700	MHCII
A780	B220
BV421	Ly6G
BV510	CD11b
BV605	CD11c
BV650	CD8

Supplementary Table 1.

The flow parameters used to run the t-SNE analyses in each specified panel.
